# Significance of forensic perpetrators with diagnosis of dementia

**DOI:** 10.1192/j.eurpsy.2025.1521

**Published:** 2025-08-26

**Authors:** L. Mužinić Marinić, I. Marinić

**Affiliations:** 1 Department of Psychiatry, Dubrava University Hospital, Zagreb, Croatia

## Abstract

**Introduction:**

Although criminal behavior is not expected in the elderly, especially those diagnosed with dementia, it is important to keep in mind the potential forensic significance of dementia. Also it is especially important to focus attention on the participation of people with dementia in the legal process, given that their cognitive impairments may impair their ability to participate in the judicial process, as well as their capacity to protect their rights and interests.

**Objectives:**

The aim of the study is to provide an overview of the forensic meaning of dementia and the assessment of persons with a diagnosis of dementia in a legal context.

**Methods:**

For the purpose of the research, a review of relevant studies in the PubMed database was performed, related to the field of forensic psychiatry of offenders diagnosed with dementia and criminal behavior of the elderly.

**Results:**

During the forensic assessment of people with dementia, it is important to assess their cognitive impairment, for which purpose various psychological tests and diagnostic processing are applied in addition to diagnosing other comorbid diagnoses, especially depression or the existence of a psychotic disorder. In dementia, an important criminogenic factor can be the use of alcohol, along with the potential for committing violent crimes.

**Conclusions:**

Depending on the severity of cognitive impairment and the existence of psychotic symptoms or a delirious state, the legal capacity of people with dementia can be significantly impaired. It is important to assess the need for security measures of psychiatric treatment, as well as the possibility of participating in the court process with regard to cognitive impairment. It is also necessary to pay attention to the protection of the rights and interests of people with dementia, as well as the ability to reason.

**Disclosure of Interest:**

None Declared

